# Supplementary figures and images for: Legionella longbeachae effector protein RavZ inhibits autophagy and regulates phagosome ubiquitination during infection
Source: PLoS One. 2023 Feb 9;18(2):e0281587. doi: 10.1371/journal.pone.0281587 (PMC9910735; doi:10.1371/journal.pone.0281587)

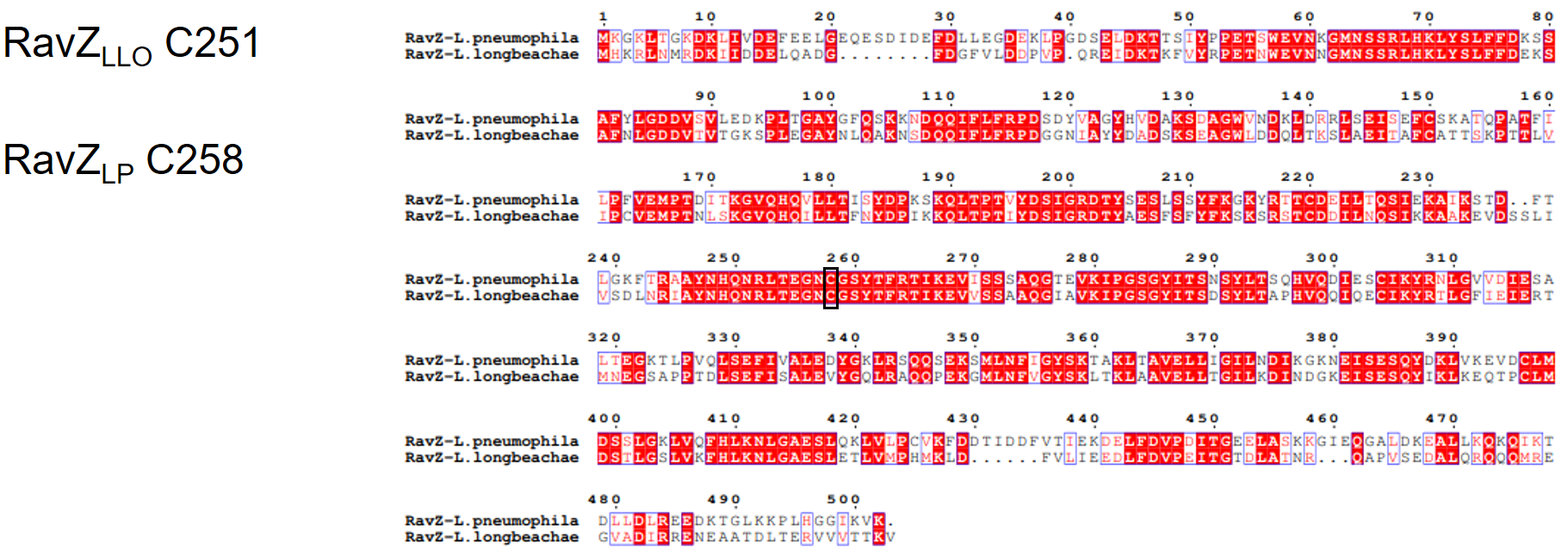

Supplement: S1 Fig — Alignment was performed by Clustal Omega (https://www.ebi.ac.uk/Tools/msa/clustalo/) and ESPript 3.0 (https://espript.ibcp.fr/ESPript/cgi-bin/ESPript.cgi). The catalytic residues Cys258 in RavZLP and Cys251 in RavZLLO are highlighted by a black box. (TIF) [file pone.0281587.s001.tif]

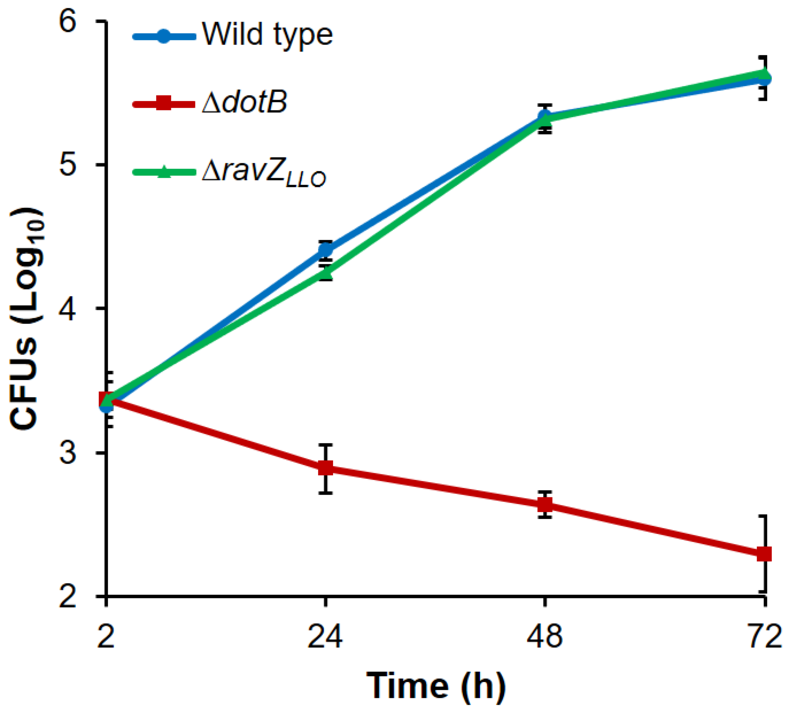

Supplement: S2 Fig — U937 cells seeded in 24-well plates were challenged with WT, ΔdotB, and ΔravZLLO L. longbeachae strains at an MOI of 10. At the indicated timepoints, infected cells were lysed by saponin and plated on CYE plates. The CFUs were counted after growing the bacteria at 37°C for 4 days. The results are from one representative experiment performed in triplicate from three independent experiments. Data are presented as the mean ± SD. (TIF) [file pone.0281587.s002.tif]

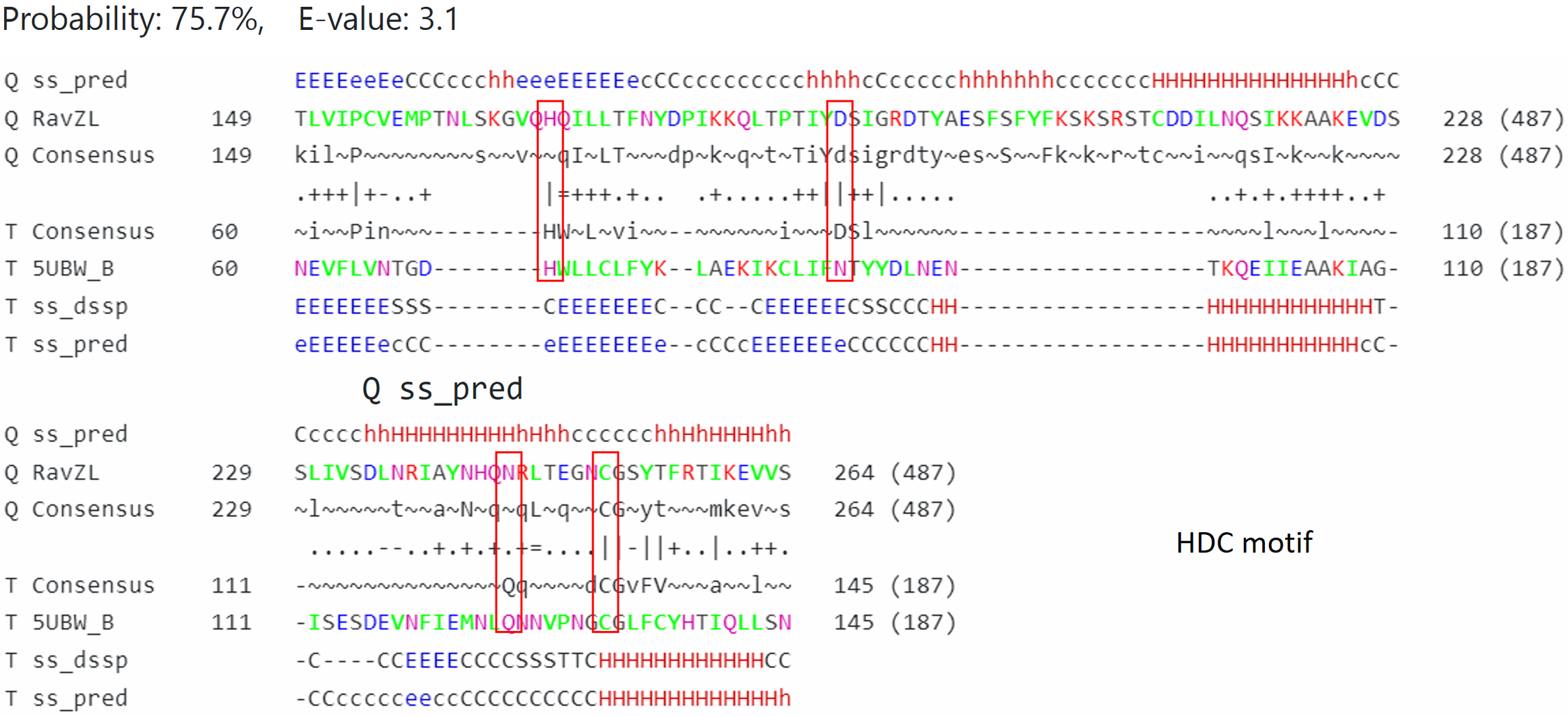

Supplement: S3 Fig — The alignment was generated by HHpred, and the catalytic His-Asp-Cys residues are highlighted by red boxes. (TIF) [file pone.0281587.s003.tif]

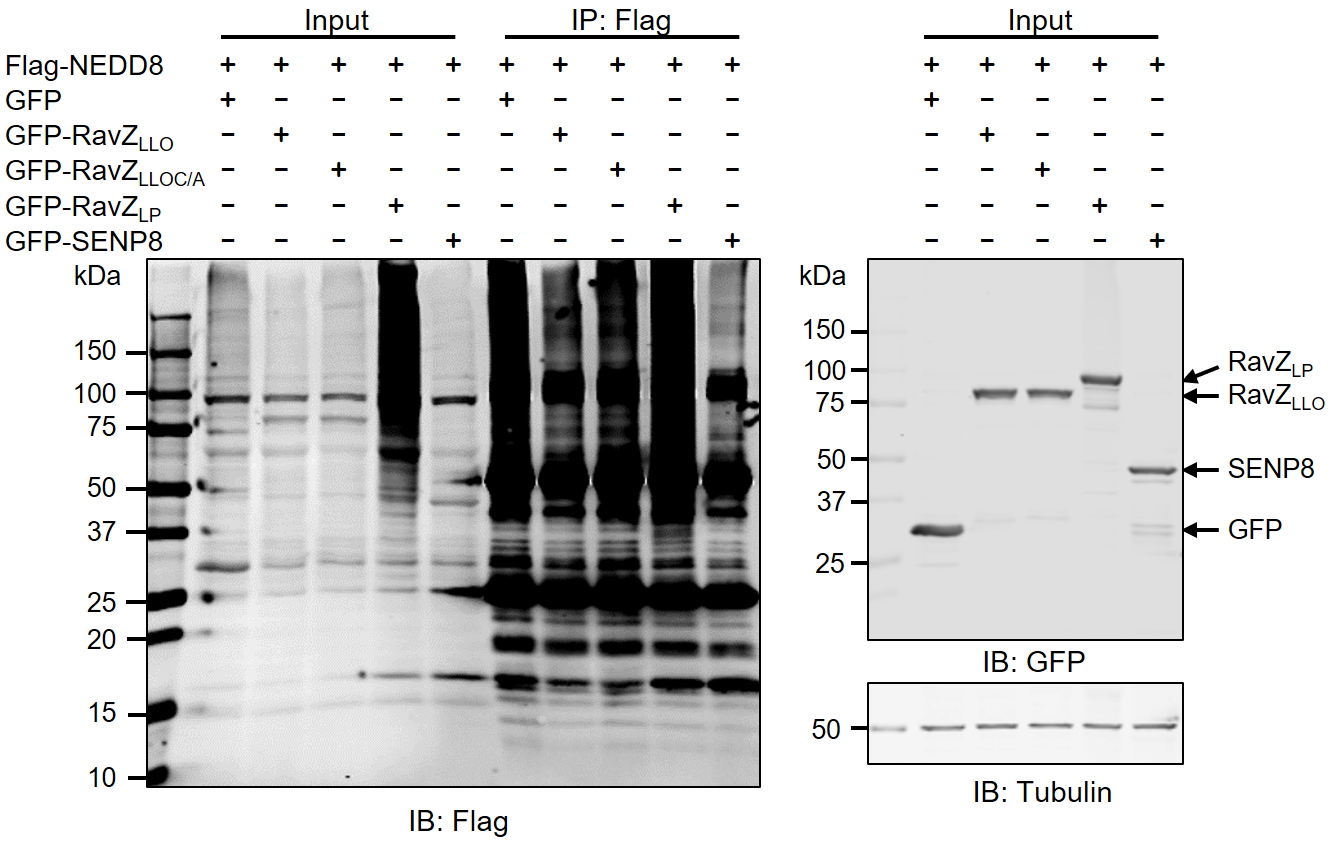

Supplement: S4 Fig — HEK293T cells were transfected to coexpress Flag-Nedd8 and each of the indicated GFP fusion proteins. At 24 h post-transfection, cells were lysed and subjected to immunoprecipitation by anti-Flag agarose. The levels of cellular polyneddylated proteins were measured by Western blot with the Flag antibody. Anti-GFP and anti-tubulin blots were performed to confirm the expression of the GFP fusion proteins and ensure equal loading of proteins, respectively. GFP-SENP8 was included as the positive polyneddylation control. The data shown are representative of three independent experiments. (TIF) [file pone.0281587.s004.tif]
