# Supplementary material for: Legionella longbeachae effector protein RavZ inhibits autophagy and regulates phagosome ubiquitination during infection
Source: PLoS One. 2023 Feb 9;18(2):e0281587. doi: 10.1371/journal.pone.0281587 (PMC9910735; doi:10.1371/journal.pone.0281587)
Supplement: S1 Table — (DOCX) [file pone.0281587.s005.docx]

**S1 Table. Bacterial strains used in the study.**

| Strains | Relevant properties | Reference |
| --- | --- | --- |
| *E. coli* |  |  |
| DH5α (λpir) | supE44 d*lacU169*(φ80*lacZ*Δ*M15)* *hsdR17 recA1 endA1 gyrA96 thi-1 relA1 pir tet::Mu recA* | Our collection |
| BL21 (DE3) | F^-^ *omp*T *hsdS*B (rB^-^ mB^-^) *gal dcm* (DE3) | Our collection |
| *L. longbeachae* |  |  |
| *L. longbeachae* | ATCC*^a^* 33462; type strain | ATCC 33462 |
| YS0001 | ATCC 33462 *L. longbeachae* serogroup 1 strain with *dotB*^-^ deletion mutation | This study |
| YS0002 | LLO+pXDC61JQ-Flag | This study |
| YS0003 | LLO *dotB*^-^+pXDC61JQ-Flag | This study |
| YS0064 | LLO Δ*ravZ_LLO_* | This study |
| YS0065 | LLO Δ*ravZ_LLO_*+pXDC61JQ-Flag | This study |
| YS0067 | LLO Δ*ravZ_LLO_*+pXDC61JQ-Flag-*ravZ_LLO_* | This study |
| YS0068 | LLO Δ*ravZ_LLO_*+pXDC61JQ-Flag-*ravZ_LLOC251A_* | This study |
| YS0069 | LLO TEM*-ravZ_LLO_* | This study |
| YS0070 | LLO *dotB*^-^ TEM*-ravZ_LLO_* | This study |
| YS0071 | LLO+pXDC61JQ-Flag-*ravZ_LLO_* | This study |
| YS0072 | LLO *dotB*^-^+pXDC61JQ-Flag-*ravZ_LLO_* | This study |
| *L. pneumophila* |  |  |
| Lp02 | Philadelphia-1 *rpsL hsdR thyA* | [1] |
| Lp03 | Lp02 *dotA*^-^ | [2] |
| Lp02 (pZL507) | Lp02+pZL507 | [3] |
| Lp03 (pZL507) | Lp03+pZL507 | [3] |
| YS0073 | LP02 Δ*ravZ_LP_* | This study |
| YS0074 | LP02 Δ*ravZ_LP_*+pZL507 | This study |
| YS0075 | LP02 Δ*ravZ_LP_*+pZL507-*ravZ_LP_* | This study |
| YS0076 | LP02 Δ*ravZ_LP_*+pZL507-*ravZ_LLO_* | This study |
| YS0077 | LP02 Δ*ravZ_LP_*+pZL507-*ravZ_LLOC251A_* | This study |
| YS0078 | LP02 TEM-*ravZ_LLO_* | This study |
| YS0079 | Lp03 TEM-*ravZ_LLO_* | This study |

*^a^* ATCC, American Type Culture Collection

**References**

1. Berger KH, Isberg RR. Two distinct defects in intracellular growth complemented by a single genetic locus in *Legionella pneumophila*. Molecular microbiology. 1993;7(1):7-19 <https://doi.org/10.1111/j.1365-2958.1993.tb01092.x>. PMID: 8382332.

2. Liu Y, Luo ZQ. The *Legionella pneumophila* effector SidJ is required for efficient recruitment of endoplasmic reticulum proteins to the bacterial phagosome. Infection and immunity. 2007;75(2):592-603 <https://doi.org/10.1128/iai.01278-06>. PMID: 17101649.

3. Duménil G, Isberg RR. The *Legionella pneumophila* IcmR protein exhibits chaperone activity for IcmQ by preventing its participation in high-molecular-weight complexes. Molecular microbiology. 2001;40(5):1113-27 <https://doi.org/10.1046/j.1365-2958.2001.02454.x>. PMID: 11401716.
