# Supplementary material for: Legionella longbeachae effector protein RavZ inhibits autophagy and regulates phagosome ubiquitination during infection
Source: PLoS One. 2023 Feb 9;18(2):e0281587. doi: 10.1371/journal.pone.0281587 (PMC9910735; doi:10.1371/journal.pone.0281587)
Supplement: S2 Table — (DOCX) [file pone.0281587.s006.docx]

| Plasmids | Properties | Reference |
| --- | --- | --- |
| pET28a | Kan^R^, *E. Coli* expression vectors for His-tagged proteins | Novagen (CAT#69864) |
| pET28a-*ravZ_LLO_* | Full length *ravZ_LLO_* in pET28a | This study |
| pSR47s | R6K suicide vector (Kan^R^, sacB) | [1] |
| pSR47s-Δ*ravZ_LLO_* | pSR47s containing the franking region of *ravZ_LLO_* | This study |
| pSR47s-Δ*ravZ_LP_* | pSR47s containing the franking region of *ravZ_LP_* | This study |
| pCMV4×Flag | For expressing N-terminal 4×Flag fusion proteins in mammalian cells | [2] |
| pCMV4×Flag-*nedd8* | *nedd8* in pCMV4×Flag | [3] |
| pCMV4×Flag-*ravZ_LLO_* | *ravZ_LLO_* in pCMV4×Flag | This study |
| pCMV4×Flag-*ravZ_LLOC251A_* | *ravZ_LLOC251A_* in pCMV4×Flag | This study |
| pCMV4×Flag-*ravZ_LP_* | *ravZ_LP_* in pCMV4×Flag | This study |
| peGFPC1 | For expressing N-terminal GFP fusion proteins in mammalian cells | Clontech |
| peGFPC1-*sdeA_Dub_* | *sdeA_Dub_* (residues 1-200) in peGFPC1 | [3] |
| peGFPC1-*ravZ_LLO_* | *ravZ_LLO_* in peGFPC1 | This study |
| peGFPC1-*ravZ_LLOC251A_* | *ravZ_LLOC251A_* in peGFPC1 | This study |
| peGFPC1-*ravZ_LP_* | *ravZ_LP_* in peGFPC1 | This study |
| peGFPC1-*senp8* | *senp8* in peGFPC1 | This study |
| peGFPC1-LC3 | microtubule-associated protein 1 light chain 3 beta (MAP1LC3B; best known as LC3) in peGFPC1 |  |
| pZL507 | For expression His_6_-tagged protein in *L. pneumophila* | [2] |
| pZL507-4×Flag-*ravZ_LLO_* | 4×Flag-*ravZ_LLO_* in pZL507 | This study |
| pZL507-4×Flag-*ravZ_LLOC251A_* | 4×Flag-*ravZ_LLOC251A_* in pZL507 | This study |
| pZL507-4×Flag-*ravZ_LP_* | 4×Flag-*ravZ_LP_* in pZL507 | This study |
| pCDNA3.1-3×HA*-ub* | Ubiquitin in p3×HACDNA3.1 | [3] |
| pXDC61m | Encodes IPTG-inducible with N-terminal TEM fusion; Cm^R^ | [4] |
| pXDC61m-*ravZ_LLO_* | *ravZ_LLO_* in pXDC61m | This study |
| pXDC61JQ | Encodes IPTG-inducible expressing N-terminal Flag fusion proteins in *L. Longbeachae*; Cm^R^ | This study |
| pXDC61JQ-*ravZ_LLO_* | *ravZ_LLO_* in pXDC61JQ | This study |
| pXDC61JQ-*ravZ_LLOC251A_* | *ravZ_LLOC251A_* in pXDC61JQ | This study |

**S2 Table. Plasmids used in the study.**

**References**

1. Xu L, Shen X, Bryan A, Banga S, Swanson MS, Luo ZQ. Inhibition of host vacuolar H+-ATPase activity by a *Legionella pneumophila* effector. PLoS pathogens. 2010;6(3):e1000822 <https://doi.org/10.1371/journal.ppat.1000822>. PMID: 20333253.

2. Sheedlo MJ, Qiu J, Tan Y, Paul LN, Luo ZQ, Das C. Structural basis of substrate recognition by a bacterial deubiquitinase important for dynamics of phagosome ubiquitination. Proceedings of the National Academy of Sciences of the United States of America. 2015;112(49):15090-5 <https://doi.org/10.1073/pnas.1514568112>. PMID: 26598703.

3. Qiu J, Sheedlo MJ, Yu K, Tan Y, Nakayasu ES, Das C, et al. Ubiquitination independent of E1 and E2 enzymes by bacterial effectors. Nature. 2016;533(7601):120-4 <https://doi.org/10.1038/nature17657>. PMID: 27049943.

4. Zhu W, Banga S, Tan Y, Zheng C, Stephenson R, Gately J, et al. Comprehensive identification of protein substrates of the Dot/Icm type IV transporter of *Legionella pneumophila*. PloS one. 2011;6(3):e17638 <https://doi.org/10.1371/journal.pone.0017638>. PMID: 21408005.
