# Supplementary material for: Legionella longbeachae effector protein RavZ inhibits autophagy and regulates phagosome ubiquitination during infection
Source: PLoS One. 2023 Feb 9;18(2):e0281587. doi: 10.1371/journal.pone.0281587 (PMC9910735; doi:10.1371/journal.pone.0281587)
Supplement: S3 Table — (DOCX) [file pone.0281587.s007.docx]

**S3 Table. Primers used in the study.**

| Primers | Sequence*^a^* | Note |
| --- | --- | --- |
| pYS1101 | CTGGGATCCATGCTGCATAAGAGATTGAAC | *ravZ_LLO_* 5F BamHI |
| pYS1102 | CTGGTCGACTCATACCTTAGTGGTTAC | *ravZ_LLO_* 3R SalI |
| pYS1103 | CTGGGATCCATGAAAGGCAAGTTAACA | *ravZ_LP_* 5F BamHI |
| pYS1104 | CTGGTCGACCTATTTTACCTTAATGCC | *ravZ_LP_* 3R SalI |
| pYS1105 | CTGAAGCTTATGATGGACCCCGTAGTCTTG | *senp8* 5F HindIII |
| pYS1106 | CTGGTCGACCTACTTTTTAGCAAGTGT | *senp8* 3R SalI |
| pYS1107 | CTGGTCGACTACCAAGCTGGTAAGA | *LLO* *dotB*^-^ knockout up SalI-F |
| pYS1108 | ATTAATTTATATACTCGAGTAAAACGAGTAGGCTCATCAGG | *LLO dotB*^-^ knockout up-R |
| pYS1109 | CCTGATGAGCCTACTCGTTTTACTCGAGTATATAAATTAAT | *LLO dotB*^-^ knockout down-F |
| pYS1110 | CTGGGATCCGGTAAGGGGGATGAAT | *LLO dotB*^-^ knockout down BamHI-R |
| pYS1111 | CTGGTCGACTCGTCATATAGAAAAACTGC | *ravZ_LLO_* knockout up SalI-F |
| pYS1112 | CGCTCCGTTAAATCTGTGGCTTCGTCGTCTATTATTTTAT | *ravZ_LLO_* knockout up-R |
| pYS1113 | ATAAAATAATAGACGACGAAGCCACAGATTTAACGGAGCG | *ravZ_LLO_* knockout down-F |
| pYS1114 | CTGGGATCCTGCGATTTGAGTGACGCACG | *ravZ_LLO_* knockout down BamHI-R |
| pYS1115 | CTGGTCGACCCAGCTCCTTCCTGGATAAA | *ravZ_LP_* knockout up SalI-F |
| pYS1116 | AATGGTTTTTTCAGACCGGTCTCATCCACTATTAATTTGT | *ravZ_LP_* knockout up-R |
| pYS1117 | ACAAATTAATAGTGGATGAGACCGGTCTGAAAAAACCATT | *ravZ_LP_* knockout down-F |
| pYS1118 | CTGGAGCTCAAATAACGTTTGTCGTACAA | *ravZ_LP_* knockout down SacI-R |
| pYS1119 | CTGAATGTATAAGAGCCAGCATTACCTTCAGTAAGGCGATTTTGATGA | *ravZ_LLOC251A_*-1 |
| pYS1120 | TCATCAAAATCGCCTTACTGAAGGTAATGCTGGCTCTTATACATTCAG | *ravZ_LLOC251A_*-2 |

*^a^* Restriction enzyme sites are underlined.
