# Supplementary material for: Legionella longbeachae effector protein RavZ inhibits autophagy and regulates phagosome ubiquitination during infection
Source: PLoS One. 2023 Feb 9;18(2):e0281587. doi: 10.1371/journal.pone.0281587 (PMC9910735; doi:10.1371/journal.pone.0281587)
Supplement: S4 Table — (DOCX) [file pone.0281587.s008.docx]

**S4 Table. Bioinformatics analysis of RavZ_LLO_ via HHpred.**

| **RavZ_LLO_** | **Target proteins** | | | | |
| --- | --- | --- | --- | --- | --- |
| Aligned region | Name | Aligned region | Probability (%) | Identities (%) | PDB ID_Chain |
| 32-486 | Uncharacterized protein RavZ, *Legionella pneumophila* Philadelphia 1 | 8-469 | 100 | 60 | 5HZY_A |
| 1-422 | *Legionella pneumophila* effector protein RavZ | 3-431 | 100 | 64 | 5MS2_A |
| 149-264 | Deubiquitinase SseL; UCH family, *Salmonella typhimurium* | 60-145 | 72.8 | 19 | 5UBW_B |
| 149-298 | SENTRIN-SPECIFIC PROTEASE 8 | 85-202 | 72.79 | 14 | 2BKR_A |
| 90-265 | *Xanthomonas* outer protein D; Enzyme, CE clan, Deubiquitinase, *Xanthomonas campestris* | 8-189 | 63.24 | 14 | 5JP1_A |
| 96-298 | Ulp1-like SUMO protease, *Chaetomium thermophilum* | 28-235 | 56.5 | 15 | 6DG4_A |
| 149-298 | Cysteine protease S273R, African swine fever virus pig/Kenya/KEN-50/1950 | 152-269 | 51.39 | 9 | 6LJ9_B |
| 149-264 | Deubiquitinase SseL Enzyme, CE clan, deubiquitinase; *Salmonella* | 211-296 | 51.39 | 22 | 5HAF_B |
| 135-300 | *Xanthomonas* outer protein D; Clan CE Family 48 Cysteine protease | 50-176 | 51.33 | 13 | 2OIX_A |
| 149-298 | RickCE; Enzyme, CE clan, deubiquitinase | 135-246 | 49.99 | 23 | 5HAM_A |
