# Supplementary material for: Legionella longbeachae effector protein RavZ inhibits autophagy and regulates phagosome ubiquitination during infection
Source: PLoS One. 2023 Feb 9;18(2):e0281587. doi: 10.1371/journal.pone.0281587 (PMC9910735; doi:10.1371/journal.pone.0281587)
Supplement: S5 Table — (DOCX) [file pone.0281587.s009.docx]

**S5 Table. *L. longbeachae* effector proteins potentially modulate host autophagy pathway.**

| ***L. pneumophila* effector** | **Known mechanism** | **Ortholog in *L. longbeachae*** | **Percent Identity** |
| --- | --- | --- | --- |
| RavZ (Lpg1683) | Cysteine protease, cleaves LC3-II | LLO_2058 | 59.4% |
| LpSpl (Lpg2176; Lpp2128; LegS2) | Degrades sphingolipids | None | - |
| Lpg1137 | Cleaves Stx17 | LLO_2404 | 62.6% |
| LegA9 (Lpg0402/Lpp2058) | Unknown | None | - |
| SidE family (SidE, SdeA, SdeB, SdeC) | Generates non-canonical ubiquitin linkages | LLO_0424 LLO_0425 LLO_0426 LLO_3092 LLO_3095 | 54.6% identity to SdeB  52% identity to SdeA  55.6% identity to SidE  53.8% identity to SdeC  54.1% identity to SdeC |
| Lgt1/Lgt2/Lgt3 (Lpg1368/Lpg1488/Lpg2862) | Glucosylates eEF1A | None | - |
| SetA (Lpg1978) | Glucosylates TFEB | None | - |
| Lpg2936 | Methyltransferase, modifies host DNA | LLO_0081 | 67.4% |
