# Supplementary material for: Legionella longbeachae effector protein RavZ inhibits autophagy and regulates phagosome ubiquitination during infection
Source: PLoS One. 2023 Feb 9;18(2):e0281587. doi: 10.1371/journal.pone.0281587 (PMC9910735; doi:10.1371/journal.pone.0281587)

Original Images for

Legionella longbeachae effector protein RavZ inhibits autophagy and regulates phagosome ubiquitination during infection

Yunjia Shi[1], Hongtao Liu[1], Kelong Ma[1], Zhao-Qing Luo[2]*, Jiazhang Qiu[1]*

[1] State Key Laboratory for Zoonotic Diseases, Key Laboratory for Zoonosis Research of the Ministry of Education, College of Veterinary Medicine, Jilin University, Changchun, China

[2] Purdue Institute for Inflammation, Immunology and Infectious Disease and Department of Biological Sciences, Purdue University, West Lafayette, IN, USA

*Corresponding authors:

E-mail: qiujz@jlu.edu.cn (JQ)

luoz@purdue.edu (Z-QL).

Figure 1B

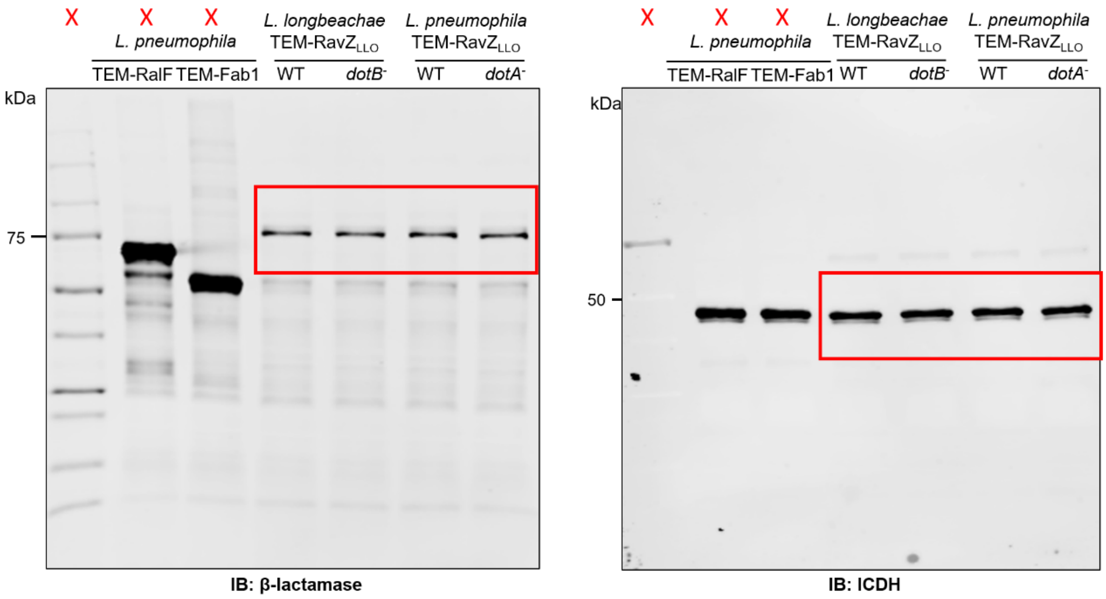

Figure 2D

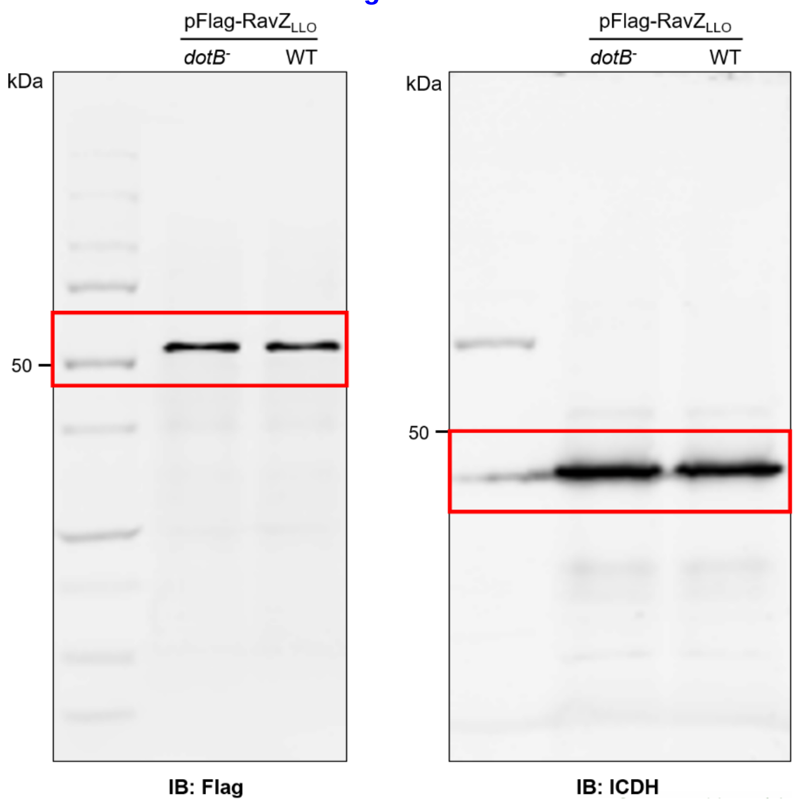

Figure 3C

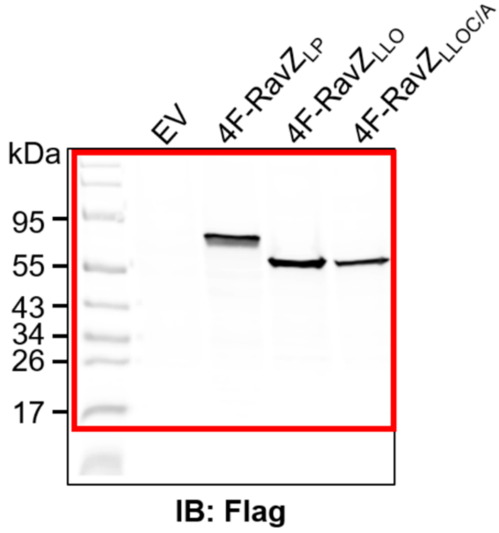

Figure 3D

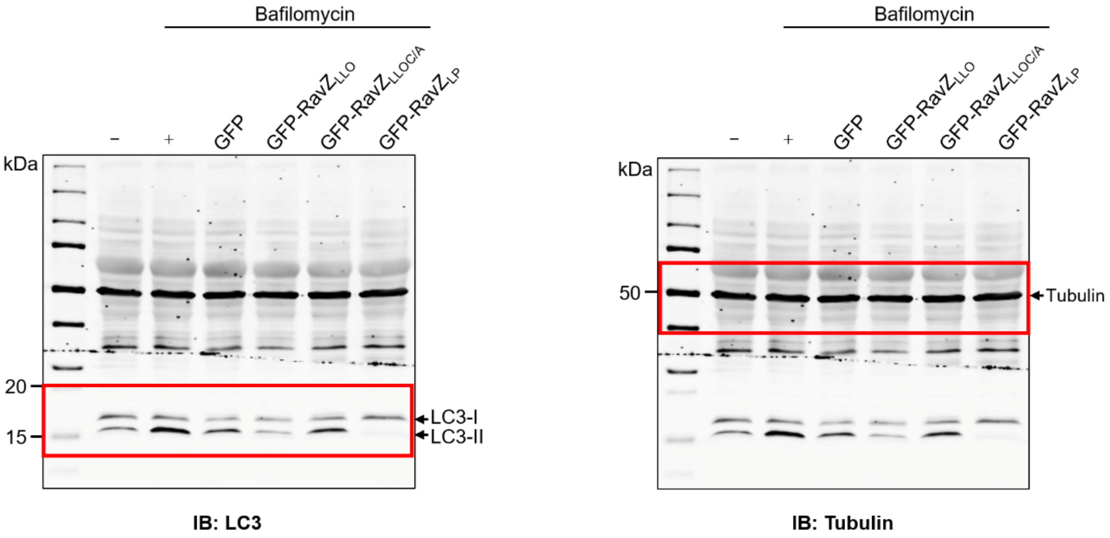

Figure 4A

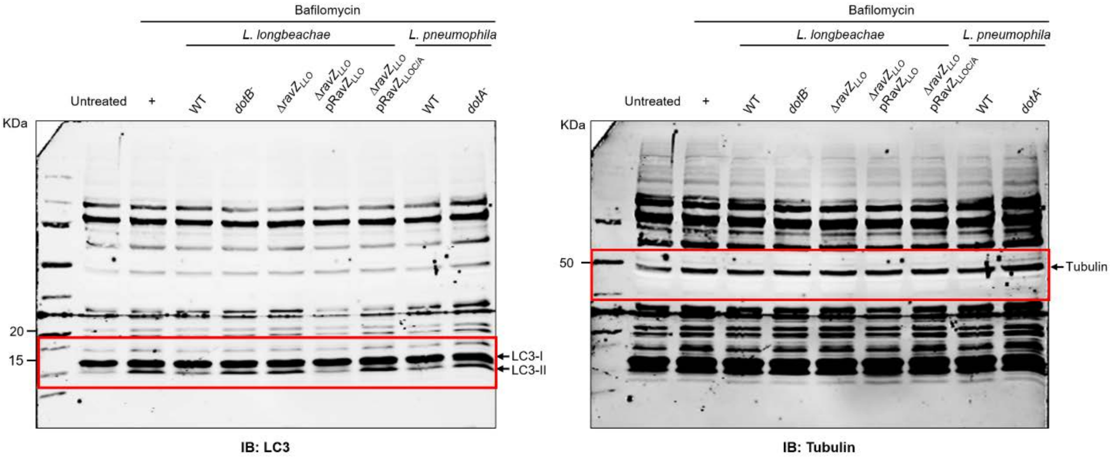

Figure 4E

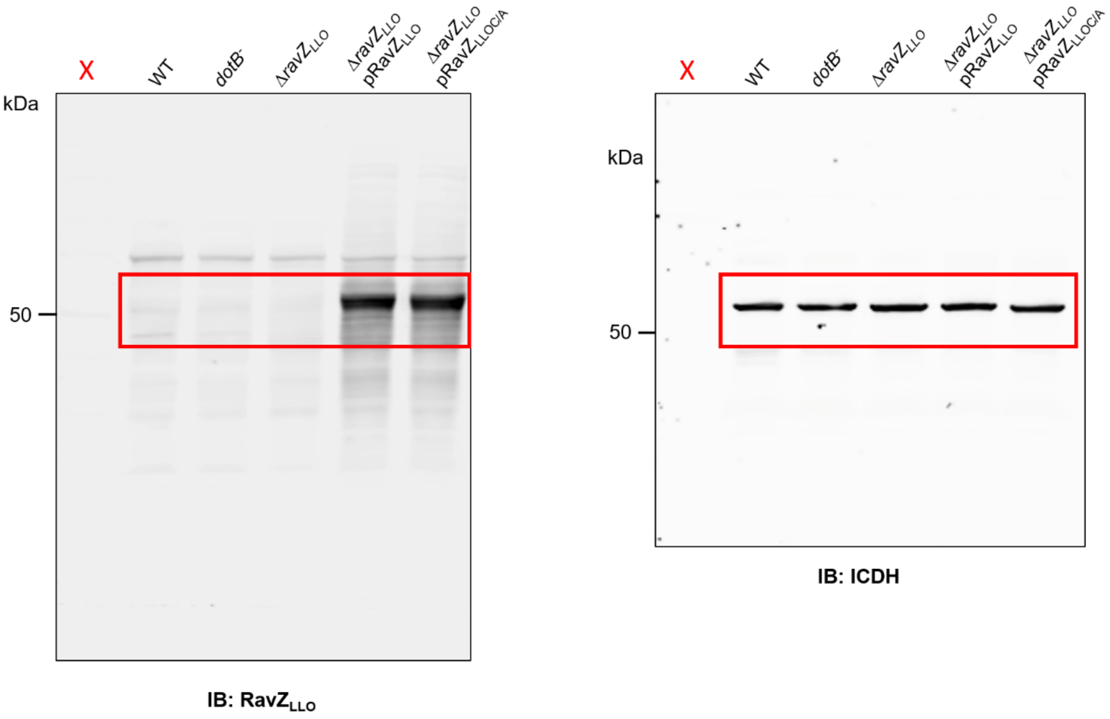

Figure 5A

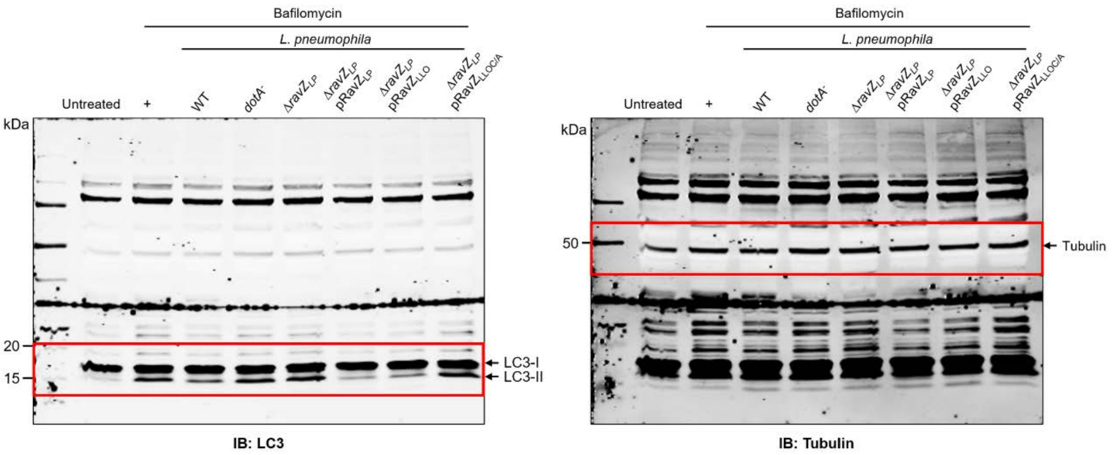

Figure 5E

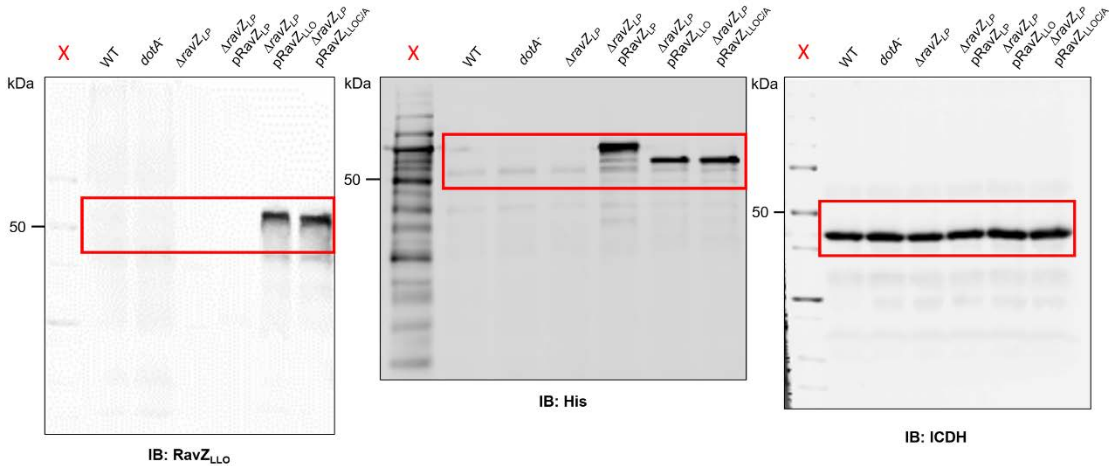

Figure 6A

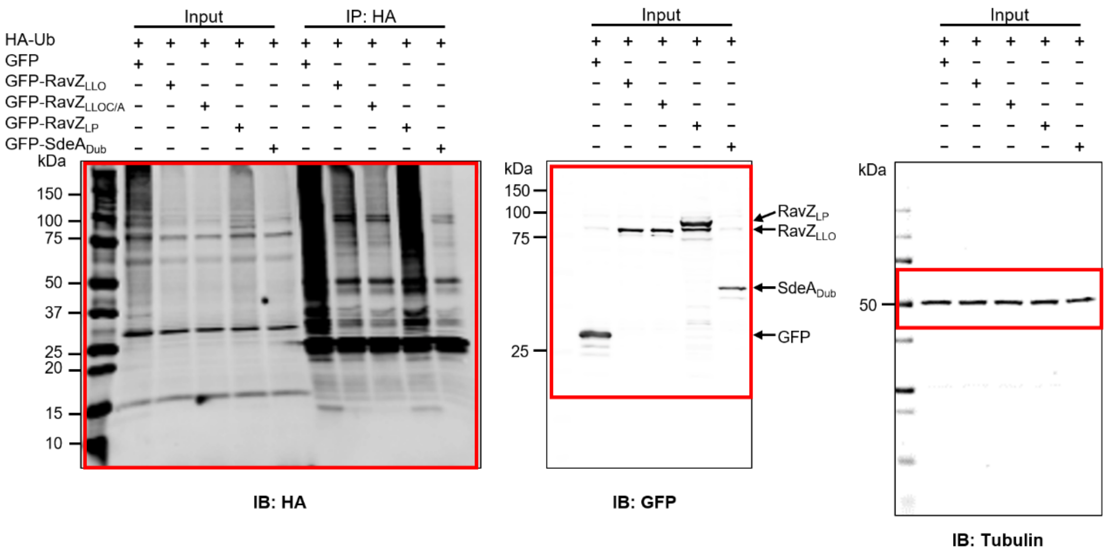

Figure 6B

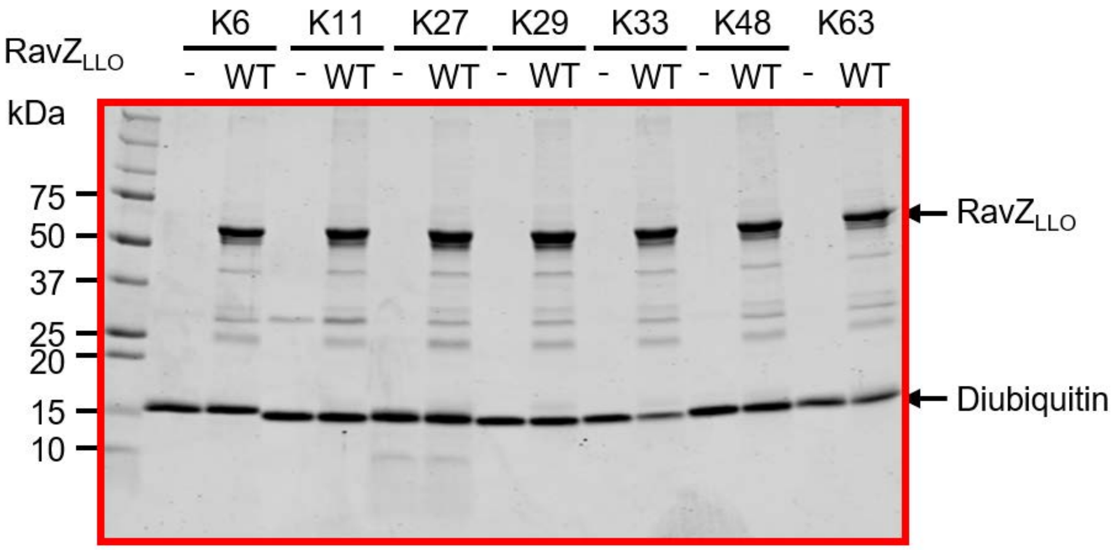

Supplementary Figure 4

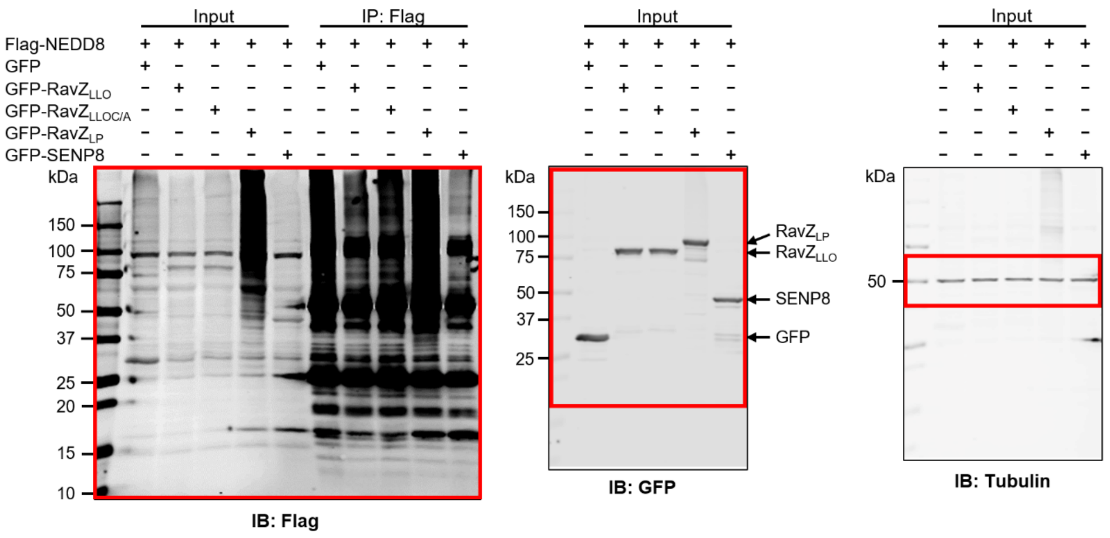

Supplement: S1 Raw images — (PDF) [file pone.0281587.s010.pdf]
