# Supplementary material for: Legionella longbeachae effector protein RavZ inhibits autophagy and regulates phagosome ubiquitination during infection
Source: PLoS One. 2023 Feb 9;18(2):e0281587. doi: 10.1371/journal.pone.0281587 (PMC9910735; doi:10.1371/journal.pone.0281587)
Supplement: Attachment — Submitted filename: Response to Reviewers.docx [file pone.0281587.s011.docx]

**Authors’ responses to comments**

Responses to Reviewer #1:

The manuscript describes the functional characterization of RavZ protein from *Legionella longbeachae* (RavZ_LLO_) and its comparison with the RavZ ortholog from *L. pneumophila* (RavZ_LP_). The authors demonstrate that RavZ_LLO_ is a secreted effector and interfere with host autophagy by removing the lipid anchor from LC3 protein, similarly to RavZ_LP_. Moreover, RavZ_LLO_ can substitute for RavZ_LP_ in *L. pneumophila*. Next, the authors show that presence in the human cell RavZ_LLO_ but not RavZ_LP_, either through direct expression or by infection with bacteria, decreases the level of polyubiquitination and polyneddylation and hypothesize that RavZ_LLO_ has deubiquitination activity. They support this view by noting that the N-terminal domain of RavZ displays fold similarity to Ubl-specific cysteine proteases, including well characterized *Salmonella Typhimurium* SseL.

1. While they observe decrease in the polyubiquitinated proteins in the human cell when RavZ_LLO_ is present, the same effect is caused by the mutant in which the active site Cys251 is replaced by Ala. In the alignment with SseL, Cys251 corresponds to active site cysteine in SseL. Moreover, no activity on various di-ubiquitins was demonstrated *in vitro*. The authors suggests that an association with an unknown host protein activated its deubiquitination activity. This is hand waving argument, and a more likely scenario is that RavZ_LLO_ stimulated deubiquitinase activity of another protein. That seems to me more reasonable explanation since RavZ_LP_ does not show this behavior, has closely similar N-terminal domain and differs in the C-terminal segment.

Response: Thanks for your suggestion. We have made the following revision. Lines 512-516: Therefore, it is possible that RavZ_LLO_ requires a cofactor to activate its DUB activity, as it is ectopically expressed in mammalian cells. Alternatively, RavZ_LLO_ might activate the DUB activity of host proteins to decrease cellular polyubiquitination and polyneddylation.

2. My other comment is to the discussion section. The first few pages repeat some of the introduction and are not relevant to the presented results, which are only discussed on pp.18-19.

Response: Thanks for your valuable suggestions. We have deleted the first two paragraph of the discussion section.
Figures:
3. Figure 2B – what is shown in the panels - a single cell or an LCV? Please indicate in the legend.

Response: Thanks for your comments. Figure 2B are the representative images showing the association of RavZ_LLO_ with the LCV. The LCVs formed in the infected cells were immunostained by anti-*L. longbeachae* antibodies. We have made the following revision. Lines 364-365: Representative LCVs with positive-association of RavZ_LLO_ during *L. longbeachae* infection.

4. Figure 2D – what is shown in fist lane?

Response: Thanks for the comment. The first lane shows the molecular weight marker (1610363, Bio-rad) in kDa.
5. Figure3A – very low resolution, not clear what the red dot (arrow?) points to. What is depicted in the panels? Multiple cells? To my eye there is little difference between the panels. What is EV, please indicate.

Response: Thanks for your advice. We apologized for the low resolution of the figures. And we have regenerated all figures at higher resolution which we hope to meet the standard. The red arrows indicate LC3 puncta (dots within the cells). The images were taken to show the effect of ectopically expressed RavZ on host autophagic responses which are represented by the formation of LC3 puncta. These images were taken to allow the inspection of multiple cells. In in cells transfected with empty vector, we can see multiple cells possessing LC3 puncta (dots) in the visual field. While in cells expressing RavZ_LP_ and RavZ_LLO_, the LC3 puncta was barely detected. EV indicates empty vector, and we have descripted EV in the revised manuscript. Please see the line 407.

As also suggested by another reviewer, we have zoomed in the dots to see clear LC3 puncta. Please see our revised Fig 3A.

6. Figure 3D – what is in first lane?

Response: Thank you for your guidance. The first lane shows the molecular weight marker (1610363, Bio-rad) in kDa.
7. Figure 4C – what is shown in the panels, full cells or LCVs?

8. Figure 5C – same question as above

Response for Q7 and Q8: Thanks. Figure 4C and Figure 5C showed the A549 cells successfully infected with *Legionella* strains. Actually, these panels show both the cell and LCV. The LCVs were immunostained with anti-*Legionella* antibodies, whereas the cells were displayed by the expression of mCherry-LC3. These representative images were used to indicate the alteration in LC3 puncta formation after infected with *Legionella*.

9. Figure 5E – no indication of expression of RavZ_LP_.

Response: Thanks for your careful review. In Fig 5E, the expression of RavZ_LP_ in *L. pneumophila* strains was determined by Western blot with the RavZ_LLO_-specific antibody. It is possible that RavZ_LLO_-specific antibody cannot recognize RavZ_LP_. Since both RavZ_LLO_ and RavZ_LP_ genes were inserted into pZL507 which contains an N-terminal His tag in the plasmid. We further probed the expression of RavZ_LP_ and RavZ_LLO_ by probing the bacterial lysates with anti-His antibodies. As shown in the following Re-fig 1, RavZ_LP_ is indeed expressed in this strain. We have added this blot into revised Fig 5E.

**Re-fig 1. The expression of RavZ_LP_ in *L. pneumophila* strains was determined by Western blot with the anti-His antibody.**

10. Figure 6A – what are the different bands in the gel image? Please mark them In the last 5 lanes there is a strong band at ~25 kDa that appears to correspond to GFP but free GFP is only indicated in the first of these lanes. What is the band around 30 kDa in the first five lanes? There are double bands in the last five lanes where we would expect RavZ that seem to be interpreted as RavZ_LP_ and RavZ_LLO_ but they should not be in the same lanes.

Response: Thanks. We guess the ~25 kDa bands you have mentioned is on the left gel of the Fig 6A. The last 5 lanes are actually anti-HA IP products. The left gel was blotted with anti-HA antibody. Therefore, the ~25 kDa bands in the last 5 lanes are actually light chain released from the anti-HA agarose and recognized by the anti-HA antibody. We have marked the left gels in our revised Fig 6A. As to the 30 kDa bands shown in the first five lanes, we believed that they are protein in the cell lysates non-specifically recognized by anti-HA antibody. We guess the double bands in the last five lanes you mentioned are the bands around ~100 kDa of the left gel. This is anti-HA blot, therefore, GFP-tagged RavZ should not be shown in this gel. One explanation for these bands is that they are unknown proteins conjugated with HA-Ub. We have revised this figure to mark the heavy chain and light chain.

11. Figure 7A, 8A – as above

Response: Thanks for your comment. Fig 7A and Fig 8A are representative immunofluorescence images showing positive-association of ubiquitinated species with the LCVs. We have revised the figure legends of Fig 7A and Fig 8A. Lines 557-560: (A) Representative images of polyubiquitinated proteins associated with LCVs. U937 cells infected for 2 h with the indicated *L. longbeachae* strains were sequentially immunostained with antibodies specific for *L. longbeachae* and polyubiquitin (FK1). Lines 568-569: Representative images of polyubiquitinated proteins associated with LCVs. U937 cells were challenged with WT *L. pneumophila*, *dotA*^-^ mutant, **∆***ravZ*, or **∆***ravZ* complemented with RavZ_LP_/RavZ_LLO_ for 2 h.

Responses to Reviewer #2:

This manuscript provides a comprehensive exploration of the role of the Dot/Icm effector RavZ during *Legionella longbeachae* infection. The *L. pneumophila* homologue is a cysteine protease that hydrolyzes lipidated LC3 to block autophagic maturation. Here the researchers demonstrate that the *longbeachae* homologue is also an effector and likely functions in the same way to suppress autophagy during infection. Interestingly the abundance of polyubiquitinated species on the LCV appears to be regulated by RavZ_LLO_ but not RavZ_LP_ indicating the possibility of a species specific function of this protein.

The study is comprehensive and scientifically sound and therefore entirely appropriate for publication in PLoS One. I have just a few minor suggestions:

1. line 65-66: while flagellin is a key difference between *longbeachae* and *pneumophila* species it doesn't seem to account for the different infection outcomes in mice (consider Massis et al - J Infect Dis - where a *pneumophila* flagellin mutant still has different outcomes to *longbeachae*.

Response: Thanks for your careful review and valuable comment. We also read the J infect Dis paper by Massis *et al*. One of the major differences between *L. pneumophila* and *L. longbeachae* is their ability to colonize the lungs of mice. While only A/J mice are permissive for replication of *L. pneumophila*, A/J, C57BL/6 and BALB/c mice are all permissive for replication of *L. longbeachae* [1, 2]. Resistance of C57BL/6 and BALB/c mice to *L. pneumophila* has been attributed to polymorphisms in Nod-like receptor apoptosis inhibitory protein 5 (naip5) allele [3, 4]. The current model states that *L. pneumophila* replication is restricted due to flagellin dependent caspase-1 activation through Naip5-Ipaf and early macrophage cell death by pyroptosis. Later on, the genomic sequencing analyses reveal that *L. longbeachae* does not encode flagella biosynthesis genes, thereby providing a possible explanation for differences in mouse susceptibility to infection between the two species [5, 6]. In Massis’s study, they found that *L. longbeachae* is more virulent than *L. pneumophila* strain lacking flagellin in a mouse model of infection, the reduced activation of the innate immunity and reduced induction of cytokines during *L. longbeachae* infection may contribute to increased bacterial colonization of the lungs, replication, and dissemination, a process that may be critical for the pathogenesis and induction of death in the infected individuals*.*

We have revised this sentence. Lines 63-65: “Unlike *L. pneumophila*, *L. longbeachae* does not encode flagella biosynthesis genes, thus partly explaining the differences in mouse susceptibility between these two species”.

2. line 81: "This apparatus comprises 25 proteins" - this is outdated given new publications Sheedlo et al and a couple of others
Response: Thanks for your valuable suggestions. We have revised our manuscript according to Sheedlo *et al*. “This apparatus comprises 27 proteins and injects a set of effector proteins across both bacterial and vacuole membranes into the cytosol of infected host cells or the vacuole lumen.” Please see the lines 81-83 in the revised manuscript.
3. Figure 3A - the quality of these IF images is unacceptable - it is impossible to determine any features within these images - they need to be higher resolution and perhaps also show a zoomed in section for the reader to deliniate LC3 puncta.

Response: Thanks for your advice. We apologized for the low resolution of the figures. And we have regenerated all figures at higher resolution which we hope to meet the standard. The images were taken to show the effect of ectopically expressed RavZ on host autophagic responses which are represented by the formation of LC3 puncta. In cells transfected with empty vector, we can see multiple cells possessing LC3 puncta (dots) in the visual field. While in cells expressing RavZ_LP_ and RavZ_LLO_, the LC3 puncta was barely detected. We have zoomed in the dot regions as your suggestion. Please see our revised Fig 3A.

**References**

1.	Asare R, Santic M, Gobin I, Doric M, Suttles J, Graham JE, et al. Genetic susceptibility and caspase activation in mouse and human macrophages are distinct for *Legionella longbeachae* and *L. pneumophila*. Infection and immunity. 2007;75(4):1933-45 https://doi.org/10.1128/iai.00025-07. PMID: 17261610.

2.	Gobin I, Susa M, Begic G, Hartland EL, Doric M. Experimental *Legionella longbeachae* infection in intratracheally inoculated mice. Journal of medical microbiology. 2009;58(Pt 6):723-30 https://doi.org/10.1099/jmm.0.007476-0. PMID: 19429747.

3.	Molofsky AB, Byrne BG, Whitfield NN, Madigan CA, Fuse ET, Tateda K, et al. Cytosolic recognition of flagellin by mouse macrophages restricts *Legionella pneumophila* infection. The Journal of experimental medicine. 2006;203(4):1093-104 https://doi.org/10.1084/jem.20051659. PMID: 16606669.

4.	Wright EK, Goodart SA, Growney JD, Hadinoto V, Endrizzi MG, Long EM, et al. Naip5 affects host susceptibility to the intracellular pathogen *Legionella pneumophila*. Current biology : CB. 2003;13(1):27-36 https://doi.org/10.1016/s0960-9822(02)01359-3. PMID: 12526741.

5.	Cazalet C, Gomez-Valero L, Rusniok C, Lomma M, Dervins-Ravault D, Newton HJ, et al. Analysis of the *Legionella longbeachae* genome and transcriptome uncovers unique strategies to cause Legionnaires' disease. PLoS genetics. 2010;6(2):e1000851 https://doi.org/10.1371/journal.pgen.1000851. PMID: 20174605.

6.	Kozak NA, Buss M, Lucas CE, Frace M, Govil D, Travis T, et al. Virulence factors encoded by *Legionella longbeachae* identified on the basis of the genome sequence analysis of clinical isolate D-4968. Journal of bacteriology. 2010;192(4):1030-44 https://doi.org/10.1128/jb.01272-09. PMID: 20008069.
